# Circulating microRNAs as Non-Invasive Biomarkers in Endometriosis Diagnosis—A Systematic Review

**DOI:** 10.3390/biomedicines12040888

**Published:** 2024-04-17

**Authors:** Arne Vanhie, Ellen Caron, Eveline Vermeersch, Dorien O, Carla Tomassetti, Christel Meuleman, Pieter Mestdagh, Thomas M. D’Hooghe

**Affiliations:** 1 Department of Development and Regeneration, KU Leuven, 3000 Leuven, Belgium; 2 Department of Obstetrics and Gynecology, University Hospital Leuven, 3000 Leuven, Belgium; 3 UGent, Center for Medical Genetics, Thent University, 9000 Ghent, Belgium; 4 UGent, Cancer Research Institute Ghen, Ghent University, 9000 Ghent, Belgium

**Keywords:** endometriosis, diagnosis, miRNA, plasma, serum

## Abstract

The aim of this systematic review is to assess the power of circulating miRNAs as biomarkers as a diagnostic tool in endometriosis. In endometriosis-suspected women with uncertain imaging, the only way to confirm or exclude endometriosis with certainty is currently laparoscopy. This creates a need for non-invasive diagnostics. We searched the literature through the PubMed database using the Mesh terms ‘endometriosis’ and ‘miRNAs’. Some, but limited, overlap was found between the 32 articles included, with a total of 20 miRNAs reported as dysregulated in endometriosis in two or more studies. MiR-17-5p was reported as dysregulated in six studies, followed by miR-451a and let-7b-5p in four studies and miR-20a-5p, miR-143-3p, miR-199a-5p and miR-3613-5p in three studies. Furthermore, a possible impact of the menstrual phase on miRNA expression was noted in five studies, while no influence of hormonal intake was observed in any included study. The modest reproducibility between studies may be attributable to biological variability as well as to the lack of universal protocols, resulting in pre- and analytical variability. Despite the identification of several suitable candidate biomarkers among the miRNAs, the need for high-quality studies with larger and well-defined population cohorts and the use of standardized protocols lingers.

## 1. Introduction

Endometriosis is a disease characterized by the presence of endometrium-like epithelium and/or stroma outside the endometrium and myometrium, according to the definition of the International working group of AAGL, ESGE, ESHRE and WES [1]. In addition, it is usually associated with an inflammatory process. The three most common manifestations are ovarian endometriomas, superficial peritoneal endometriosis and deep pelvic endometriosis, with the latter being signified as invading 5 millimeters below the peritoneal surface. Other forms include bowel endometriosis, bladder endometriosis, extra-abdominal endometriosis and iatrogenic endometriosis [1,2].

The disease can either occur asymptomatically or be accompanied by symptoms such as chronic pelvic pain, dysmenorrhea, dyspareunia, dyschezia and sub- or infertility. Nevertheless, the severity of the symptoms does bear a weak correlation to the extent and location of the endometriosis [3]. Given this variation in intensity and the non-specificity of the symptoms, the diagnosis is often delayed: on average only 7 to 11 years after the onset of the first symptoms [4]. Suspection of endometriosis is usually based on symptoms and clinical examination, with confirmation by imaging and, if required, laparoscopy. Ultrasound and MRI are the preferred imaging methods used to detect ovarian cystic endometriosis [5]. However, when this imaging is negative, endometriosis cannot be excluded. In case of the suspicion of endometriosis and negative imaging, a diagnostic laparoscopy is recommended. This can detect endometriosis when it is not seen on imaging, such as superficial endometriosis [6]. During laparoscopy, biopsies can be taken to obtain histological confirmation [2]. This is also necessary to distinguish cystic ovarian endometriosis from ovarian cancer, which cannot be differentiated on imaging [5].

Endometriosis affects about 2 to 10% of all women of reproductive age, 50% of infertile women and even 2 to 5% of postmenopausal women [7,8]. The therapy depends very much on the symptoms, the extent and the type of endometriosis and has to be considered case by case. Treatment is conducted either symptomatically, with suppression of symptoms with NSAIDs or hormonal treatment, or the endometriosis is removed surgically, preferably by laparoscopy [7].

Given its high prevalence and the well-known diagnostic delay in endometriosis, many women suffer from debilitating symptoms over many years. Therefore, a non-invasive diagnostic test has been a research priority for the scientific community for decades [9]. During this period, hundreds of studies have been conducted on this topic; however, no non-invasive diagnostic test has reached the accuracy required for use in clinical practice. In 2016, a Cochrane series of systematic reviews was published on this topic, presenting an overview of all molecules that have been studied [10]. However, no non-invasive diagnostic test achieved the accuracy required for clinical practice. CA-125, as a marker of peritoneal inflammation, was found to be elevated only in advanced stages of endometriosis, and it has low specificity [11]. In the case of cytokines, for example, IL-6, measurements are highly dependent on the method of sample collection, and the short half-life and circadian rhythm must be taken into account. Endometrial nerve fibers were also examined, using protein gene product 9.5 (PGP9.5) as a marker. This came out as promising, but mainly the specific sample collection and the difficult immunohistochemistry assays limit it as a good diagnostic biomarker at this time [11].

As described in ‘Research Priorities for Endometriosis’ (2017) [12], the search for biomarkers for endometriosis remains a number one focus, with microRNAs (miRNAs) being one of the primary targets in recent years. The latter are small (19 to 24 nucleotides long), non-coding, single-stranded RNAs, whose function is to regulate post-translational gene expression [13]. They achieve this by binding to their complementary messenger RNA (mRNA). This leads either to the repression of translation of the mRNA or the cleaving of the mRNA. Both processes result eventually in post-transcriptional gene silencing. As such, miRNAs affect cellular apoptosis, differentiation and proliferation [14].

These processes are also very important in the development and persistence of endometriosis [15], which is why an association is currently being investigated between the onset of this condition and specific miRNAs. The investigation of miRNAs in blood is minimally invasive, and moreover, miRNAs in the circulation are stable and easily accessible. Therefore, they could serve as a promising diagnostic biomarker [16]. However, despite the increase in interest and research conducted in recent years, currently little overlap exists between studies [17]. It is for this reason that we performed this systematic review to summarize the most differentiated miRNAs in the circulation of patients with endometriosis and hence the best candidate biomarkers.

## 2. Materials and Methods

**Figure 1:**
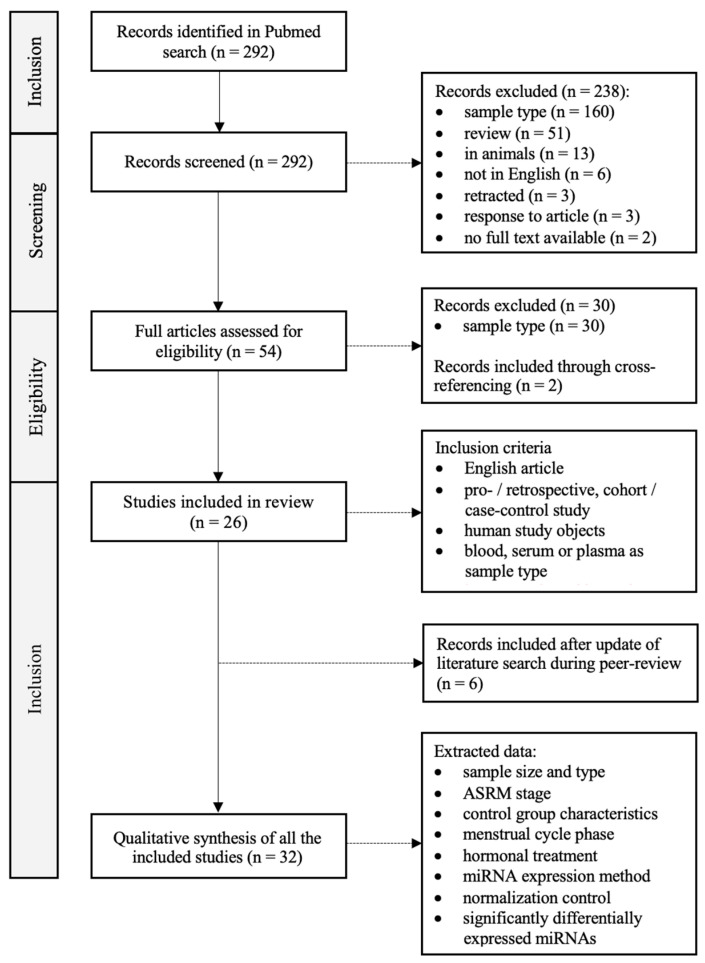
PRISMA flowchart of literature search and study selection.

We performed a systematic review according to the PRISMA guidelines (registration code: 202410066) [18]. The PubMed database was searched using the Mesh terms ‘Endometriosis’ and ‘MicroRNAs’, with inclusion up to 17 November 2022. Titles, abstracts and full papers were individually assessed for eligibility by two authors (AVH and EC). A flow chart of the literature search is provided in Figure 1. A total of 292 articles were found. All titles and abstracts were carefully read and assessed. After this initial screening, 54 articles were sought for retrieval and assessed for eligibility. Inclusion criteria were the following: (1) article available in English; (2) humans as study subjects; (3) miRNA analysis performed in whole blood, plasma or serum; (4) prospective, retrospective or case–control studies; (5) published in a peer-reviewed journal. Any ASRM stage [19] of endometriosis, any menstrual stage and any technique for miRNA expression analysis were allowed.

Considering these conditions, a selection of 26 articles fulfilled all inclusion criteria and hence were considered appropriate for our study. Reasons for exclusion were the following: (1) article only available in Chinese language; (2) animals as study subject; (3) study performed in vitro; (4) review article; (5) correction of previous article or response to existing article; (6) retracted article. The 26 included articles were read in detail and the following data were extracted: the number of cases and controls, the ASRM stage, the characteristics of the control group, the sample type (plasma or serum), the phase in the menstrual cycle, the hormonal treatment, the procedure applied for miRNA expression analysis, and the normalization control used (Table 1). During peer review, an update of the literature search was conducted, with the incorporation of 6 additional articles that met the inclusion criteria. The final date of inclusion was 4 November 2023, resulting in a total of 32 articles.

## 3. Results

A total of 32 studies were identified through PubMed and included in this systematic review (Figure 1, Table 1). Altogether, there were 1527 patients with confirmed endometriosis. The diagnosis was made using MRI and/or laparoscopy or laparotomy, and it was confirmed histologically where possible. Fourteen studies included patients with endometriosis stage I to IV based on the ASRM classification [20,25,26,28,30,31,34,38,39,40,41,42,43,49]. Women with moderate-to-severe endometriosis (stage III to IV) were included in ten studies [22,23,27,37,44,45,47,48,50,51]. Six studies did not mention the stage of endometriosis [29,32,33,35,36,46], and the remaining two studies included only women with stage I to II [24] and II to IV [21], respectively. The number of control patients across all studies was 1167 in total. The control groups consisted of self-reported healthy women or patients with symptoms suggestive of endometriosis which could not be confirmed or women with other benign gynecological conditions. The majority of the studies included patients with no use of hormonal treatment in the last 3 months [20,22,23,24,25,26,27,31,32,34,35,37,38,42,43,45,47,50,51]; Gu et al. [46] and Bahramy et al. [48] even decided to not include patients who took hormonal treatment during the last 6 months. Ten studies did not specify the hormonal treatment of the included patients [21,29,30,31,33,36,39,40,44,49]. A diverse study population was used in Pateisky et al. [41], Nisenblat et al. [42] and Moustafa et al. [28], with both women who were taking hormones at the time of the study and women who were not taking them, as well as patients in whom this was uncertain. The menstrual phase was not specified in seventeen studies [20,21,22,26,29,30,31,33,34,35,36,39,40,44,45,49,51]. Eleven studies made a distinction between the proliferative phase and the secretory phase [23,24,25,27,28,37,38,41,46,47,48]. Nisenblat et al. [42] and Vanhie et al. [43] even distinguished a third phase: the menstrual phase. Only women in the secretory stage were included in the studies by Gu et al. [46] and Tahermanesh et al. [50], while Hu et al. [27] and Neuhausser et al. [32] solely collected samples in the proliferative phase. Altogether, 471 patients were in the secretory phase at the time of sampling, while 519 patients were in the proliferative phase and 38 in the menstrual phase.

Sixteen studies investigated the presence of miRNA in serum [20,21,22,23,24,25,26,27,28,29,30,31,32,33,34,35], and an equal number of studies did so in plasma [36,37,38,39,40,41,42,43,44,45,46,47,48,49,50,51]. In some cases, the selection of miRNAs was conducted based on a literature review of existing studies [22,25,26,27,28,29,30,31,32,34,35,39,40,44,47,48,51]. Two studies used online databases for targeted analysis to predict miRNAs’ target genes [33,50]. In other cases, the presence of miRNAs was screened by microarray [20,21,23,27,37,41,42,46], RT-qPCR [36,38] or NGS [24,43,45,49]. Validation was primarily conducted with RT-qPCR. Various normalization controls were used, however, U6 snRNA was the most prevalent in thirteen studies [20,22,23,25,26,27,28,30,31,32,34,35,50]. An independent validation test was performed in a mere seven studies [32,33,41,42,43,45,51]; Bendifallah et al. [49] worked with internal cross-validation.

**Figure 2:**
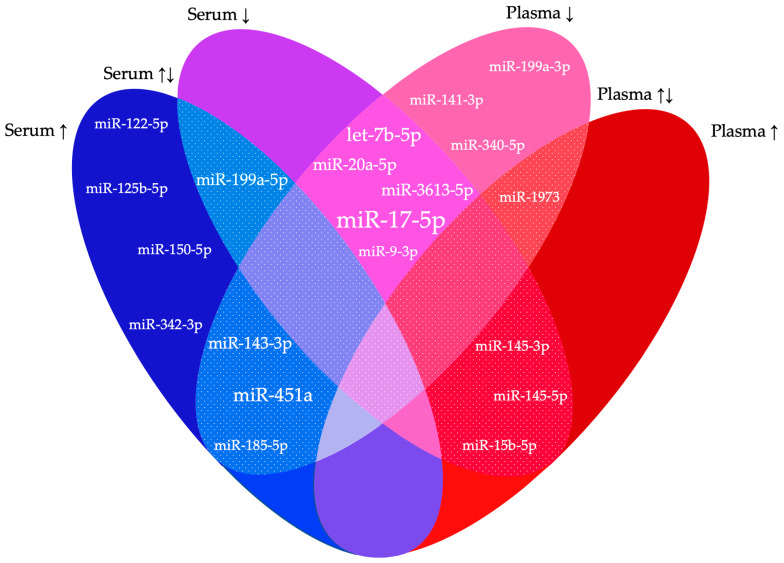
Significantly dysregulated miRNAs in two or more studies.

Across all studies, a total of 141 miRNAs were significantly differentially expressed between endometriosis patients and controls. However, a mere 20 were reported in at least two studies, while the remaining 121 could not be replicated in more than one study (Table 2, Figure 2). Similar results were obtained in the studies of Cosar et al. [23] and Moustafa et al. [28], both conducted in serum. One miRNA was downregulated in both studies (hsa-miR-3613-5p), as in the study of Walasik et al. [51], and four miRNAs were upregulated (hsa-miR-125b-5p, hsa-miR-150-5p, hsa-miR-342-3p and hsa-miR-451a). The latter was also found to be elevated in a third study from Nothnick et al. [25], but it was found downregulated in a fourth study of Walasik et al. [51]. Wang et al. [20] and Maged et al. [26] found matching results in serum, with both reporting the occurrence of hsa-miR-122-5p as downregulated. miRNAs described as decreased in plasma in two studies were hsa-miR-141-3p, hsa-miR-199a-3p and hsa-miR-340-5p [38,42,45,46,48]. Even in different media (plasma and serum), similar changes in miRNAs were found, with decreased hsa-miR-9-3p in Wang et al. [20] and the selection cohort of Nisenblat et al. [42]. Conflicting results for dysregulated miRNAs in two studies were found for four miRNAs. Hsa-miR-145-3p was found to be elevated in serum [20], while the opposite was found in plasma [42]. Wang et al. [24] described hsa-miR-15b-5p as downregulated and hsa-miR-185-5p as upregulated, in contradiction to Suryawanshi et al. [36] and Hossein Razi et al. [44], respectively. Hsa-miR-1973 occurred both elevated and decreased in plasma according to Suryawanshi et al. [36] and Bendifallah et al. [49] respectively. Four miRNAs were listed as differentially expressed in endometriosis in three studies: hsa-miR-451a, hsa-miR-199a-5p and hsa-miR-20a-5p [20,21,23,24,25,26,28,37,45]. The first two were found to be dysregulated in three studies conducted in serum, with hsa-miR-451 being consistently upregulated [23,25,28] and hsa-miR-199a-5p being both up- and downregulated [20,21,26]. A recurrently declined expression in different media was detected for hsa-miR-20a-5p. For hsa-miR-143-3p, a consistent increase was found in serum by Cosar et al. [23] and Kumari et al. [33], and a decrease was seen in plasma by Papari et al. [45]. Two miRNAs occurred in four studies. Hsa-miR-451a was discussed above. The second, hsa-let-7b-5p, had a reduced presence in endometriosis patients in both two plasma [45,46] and two serum studies [22,28]. Hsa-miR-17-5p was even documented as being consistently decreased in six studies, including three studies in plasma [37,40,45] and three in serum [29,34,35].

No influence of the menstrual phase on miRNA expression was found in eight studies [20,23,24,28,38,45,47,48]. This contradicts five other studies, which did find cyclic variation in miRNAs in the circulation of endometriosis patients [22,25,41,42,43]. Also, three studies did mention the menstrual phase of their study population, yet they discussed no further impact of this on miRNA levels [27,37,46]. Three studies investigated the presence of a correlation between miRNA differentiation and hormonal use [28,41,43], and all detected no significant interaction between hormonal therapy and miRNA expression.

This Euler diagram shows the differentially expressed miRNAs reported in two or more of the included studies. For both serum and plasma, it is displayed whether the miRNAs were up- (↑) or downregulated (↓) in the different studies. The speckled sections represent regions of conflicting results. The larger the font of the miRNAs, the more often they were described in the included studies.

## 4. Discussion

This systematic review combines the findings of 32 studies on circulating miRNAs as potential biomarkers for endometriosis. All combined, 1527 patients and 1167 controls were included, and a total of 141 miRNAs were reported as differentially expressed. Overall, we found limited overlap and conflicting results in the differentially expressed miRNAs between the included studies: only one miRNA was consistently differentially expressed in serum and plasma in at least six studies (hsa-miR-17-5p), with hsa-miR-451a and hsa-let-7b-5p in four studies, hsa-miR-20a-5p, hsa-miR-143-3p, hsa-miR-199a-5p and hsa-miR-199a-5p in three studies and all other miRNAs in fewer studies.

MiR-17-5p seems to be a promising diagnostic biomarker as it was reported in six independent studies as downregulated in endometriosis patients. However, when looking at the largest studies (with inclusion of 100 or more patients and controls) [28,30,34,38,40,42,43,49], only in two [34,40] of these seven studies was miR-17-5p differentially expressed in endometriosis. It might be possible that miR-17-5p is a sensitive outlier (easily very highly up- or downregulated) and is therefore prone to be found differentially expressed in smaller study populations. To further clarify this, follow-up studies are needed with large study populations or new studies with validation cohorts to confirm these results.

### 4.1. Function of the Most Frequently Reported miRNAs

The most documented differentiated miRNA in endometriosis was miR-17-5p, and it was described in as many as six studies, all of which reported it as consistently decreased in endometriosis patients. As a consequence of its downregulation, the suppressive effect on its target genes declines, resulting in higher levels of hypoxia-inducible transcription factors (*HIF-1a*) and vascular endothelial growth factor (*VEGF-A*), among others [37]. This induces hypoxia, inflammation and angiogenesis with cell proliferation, migration and invasion, all most presumably involved in the pathogenesis of endometriosis [37,45].

Let-7b-5p was reported in four different studies. All these studies obtained the same results: a reduced level of this miRNA in subjects with endometriosis. Due to the lower expression of the let-7 family, higher *KRAS* levels could arise with an increase in cell proliferation and invasion [52]. The possible involvement of let-7b in endometriosis pathophysiology was also supported by a study conducted in mice [53]. This showed that when injecting let-7b intraperitoneally, the expression of genes implied in the pathophysiology of endometriosis decreases, as well as the size of endometriosis lesions [53].

MiR-199a-5p was found to be reported as significantly dysregulated in three included studies. Hsu et al. [21] observed a significant decrease in this miRNA in women with endometriosis in comparison with controls. By contrast, Maged et al. [26] and Wang et al. [20] reported it as upregulated. The latter additionally found significantly higher miR-199a-5p expression in advanced endometriosis (grade III-IV) compared to the less progressed forms (grade I-II) [20]. This suggests a possible correlation between endometriosis severity and the level of miR-199a-5p. The function of this miRNA similarly indicates its involvement in the pathogenesis of endometriosis. It targets genes involved in apoptosis, cell proliferation and hormone-mediated signaling pathways [20]. Moreover, miR-199a-5p suppresses the IkappaB kinase beta/nuclear factor-kappa B pathway and inhibits interleukin-8 (IL-8) production in endometrial stromal cells (ESCs), enhancing the adhesion, migration and invasiveness of ESCs [54].

### 4.2. Lack of Reproducibility 

With the increase in research on miRNAs as biomarkers in endometriosis in recent years, several miRNAs have been discovered that show a significant increase or decrease in this disease. Unfortunately, little overlap exists between different studies. This may be due to different biological as well as pre-analytical and analytical factors.

#### 4.2.1. Biological Variability

Considerable inter- and intra-individual biological variability exists in endometriosis. In general, the condition itself occurs in several forms, with superficial peritoneal, deep pelvic and ovarian endometriosis being the three most frequent ones [1]. Symptoms similarly vary widely, from pelvic pain, infertility and dyschezia to no symptoms at all. Moreover, these symptoms show poor correlation to the form of the endometriosis or its extensiveness. This makes timely recognition and diagnosis of the condition challenging [3]. On top of that, a big difference is seen in response to the treatment chosen [55].

Considering endometriosis is an estrogen-influenced disease, biomarkers may fluctuate depending on the menstrual phase [16]. Here, eight studies have not found any menstrual influence on the miRNA profile in endometriosis [20,23,24,28,38,45,47,48]. This is in contrast to five studies that did describe the cycle phase as a confounder [22,25,41,42,43]. Cho et al. [22] reported an increase in the expression of four miRNAs of the let-7 group in endometriosis patients, while the expression levels in the control group remained stable. Furthermore, the circadian rhythm was also found to influence miRNA expression. A reduced expression of three miRNAs was detected by Rekker et al. [38] in morning samples compared to evening samples, in both endometriosis and non-endometriosis patients.

The included studies show great diversity in the composition of the control groups: self-reported healthy women, women undergoing laparoscopy for various gynecological conditions and women with endometriosis-like symptoms but a negative laparoscopy. The characteristics of the included patient population must be comparable to those of the population in which the non-invasive test would be applied in the clinic. In this case, that means imaging-negative women with endometriosis-like symptoms, where the non-invasive test would be used either to complement imaging or to replace expert imaging. Additionally, it is important to properly describe the patients’ profile, as ethnicity [16], physical activity [16], BMI [41] and hormonal intake [56] might also affect miRNA expression. However, the influence of the latter was investigated in three included studies, in which none showed a difference in the occurrence of miRNAs [28,41,43]. By contrast, a study by Dabi et al. [56] did show a significant increase in miR-548p and miR-5481 levels under hormonal intake in comparison with no hormonal use. The World Endometriosis Research Foundation (WERF) created guidelines related to patient selection and sample collection in order to allow for large-scale collaborations and to establish higher homogeneity [57,58].

#### 4.2.2. Pre-Analytical Variability

Pre-analytical variability refers to the differences in results attributable to sample collection, processing and preservation. Cheng et al. reported 72% of differences in miRNA expression originating from variations in processing [59]. For instance, plasma contains a variable, processing-dependent amount of residual platelets, microvesicles and cellular debris, which cause differences in miRNA level measurements. To minimize these differences between serum and plasma, it is important to determine the residual platelets in the sample, and additional centrifugation or filtration is recommended [59]. More specifically, in order to obtain platelet-free plasma (PFP), samples should be centrifuged at 60,000 g*min, discarding 99% of the platelets. However, despite the additional removal of platelets from plasma, platelet microvesicles (PMVs) still affect the miRNA profile. These are released during platelet activation in plasma processing. It is therefore essential to minimize this activation, for example, by choosing a proper anticoagulant for sample collection [60].

Ethylenediaminetetraacetic acid (EDTA) is frequently used for sample collection in studies on miRNA detection [42,45,49]. However, Mussbacher et al. [61] found citrate-theophylline-adenosine-dipyridamole (CTAD) to be superior, as it showed the lowest degree of in vitro platelet activation. An increase in miRNA levels in plasma was observed when EDTA was used compared to CTAD. Additionally, this increase was time-dependent, and it was more pronounced when stored at room temperature versus lower temperatures of 4 °C [61]. Also, Zhelankin et al. [60] observed a higher miRNA percentage in EDTA samples (80%) compared with CTAD samples (45%), most likely due to higher hemolysis in the EDTA samples. Consequently, the use of EDTA as an anticoagulant could lead to higher miRNA levels, though with lower miRNA diversification as well as biased miRNA detection caused by platelet activation and/or hemolysis [60]. The influence of hemolysis on the miRNA profile is especially seen for miRNAs found in higher concentrations in erythrocytes, such as miRNA-16 [62]. Hence, some miRNAs, like miRNA-122, are little to not affected by hemolysis, making this kind of variability miRNA-specific [62]. By determining the hemolysis index, a lipemia-independent measurement of hemolysis, the degree of hemolysis can be taken into account to correct for its influence on the miRNA profile [63].

As mentioned above, the temperature of sample preservation as well as the period from collection to processing can affect the miRNA profile. Kupec et al. [64] found a slightly decreased detection value in the included sixteen miRNAs after storage for 14 days at −80 °C, compared to analysis within 24 h at a temperature of 4°. However, this decrease was significant for only one miRNA. Matias-Garcia et al. [65] detected no significant changes in miRNA expression of eight selected miRNAs, not even at temperatures of −180 °C and up to 17 years of storage. Similarly, seven of the eight miRNAs remained stable at one to four freeze–thaw cycles. One miRNA experienced a significant downregulation only starting from cycles three and four. These results encourage the use of biobanks, which allow for multiple samples to be stored for longer periods without affecting the stability of the miRNAs [65].

Additionally, RNA isolation similarly can cause pre-analytical variability. A variety of kits and different techniques exist for this, e.g., based on columns, beads or chemicals [66], with the column-based isolation method repeatedly described as most effective [66,67]. The miRNAs can be isolated as a fraction of total RNA, as an enriched fraction of small RNAs or packaged in extracellular vesicles. Moreover, the amount of starting material is important as well: little input material leads to little miRNA isolation, too much material in turn leads to rapid saturation and a reduction in extraction efficiency [66]. The included studies in this systematic review mainly used the MirVana RNA Isolation Kit, Trizol LS reagent and the miRNeasy Mini Kit. Interstudy discrepancies in miRNA profiles may be partly attributable to the predominant isolation of certain miRNAs in each of the used protocols and miRNA extraction kits [66].

Lastly, saliva-based miRNAs are also potential diagnostic markers in endometriosis. Consideration does need to be given to the varied salivary composition, with cytokines, antibodies, hormones and antimicrobial elements. Diet and oral hygiene may also influence saliva composition, quality and examination [68]. Beyond that, this medium offers several advantages, such as cost-effectiveness, non-invasiveness and easy sample collection [69]. On top of that, salivary miRNAs frequently occur encased in exosomes, which enhances their stability [68]. In a recently published study, Bendifallah et al. [70] found a possible saliva signature consisting of 109 miRNAs. In this interim analysis of an external validation study, a combination of NGS and artificial intelligence was used and an AUC of 96.7% was obtained. However, the small sample size of 200 patients and the missing analysis on follow-up data must be taken into account. Further studies are needed to confirm these results.

#### 4.2.3. Analytical Variability

Analytical variability denotes the variability in data resulting from different methods of sample analysis. These mainly depend on the miRNA profiling platform adopted, with micro-array, RT-qPCR and NGS being the most often applied. The latter is able to quantify a whole transcriptome through RNA sequencing [71]. Only a small amount of minimal starting material of <10 ng and possibly even as little as 1 ng is needed for this. Furthermore, NGS can identify isomiRs, which are miRNAs with other sequences than their reference sequence in miRBase [66]. High-throughput screening is similarly possible by using (hybridization-based) microarrays, which can detect thousands of non-coding RNAs at once. This contrasts with qRT-PCR, which has a low processing power. Nevertheless, it is a fast, sensitive and accurate method with only a small amount of input material required [71]. Therefore, NGS and microarrays are principally applied in the discovery phase [72], while qRT-PCR is primarily used as the gold standard in the validation of screened miRNA candidates [72].

Another limitation of the analysis process is the absence of a standardized normalization strategy in RT-qPCR [73]. The aim of normalization is to obtain the most reliable and reproducible result possible by reducing technical bias. Various normalization methods have been created with both the use of exogenous and endogenous oligonucleotides [73]. Cell-miR-39 and cell-miR-45, sourced from *Caenorhabditis elegans*, are the most frequently used synthetic miRNAs [66]. These spike-ins can be introduced in precisely defined concentrations of biological samples, either before RNA isolation or before reverse-transcription, to adjust for variability in these processes. However, this approach does not allow for controlling the quality of input material [66].

Only one of the included studies opted for an exogenous normalization control, this was Wang [24] with cell-miR-39. All other studies worked with endogenous reference controls, with about slightly over one-third of the studies using U6 snRNA. Nonetheless, this snRNA has been found to exhibit a great variability in expression in both healthy people and people with certain pathologies [66]. On top of that, it similarly does not reflect the same biological properties as miRNAs and has been shown to be unstable in the circulation [73]. Another frequently described endogenous normalization control is miR-16, seen here in three studies. As described above, it is highly expressed in erythrocytes, leading to higher concentrations in samples as a result of hemolysis [62]. Furthermore, it is used as a biomarker in various diseases [66], and its levels are modifiable by inflammation and stress [45]. This brings into question its use as a reference control. One other applied method that would yield the greatest decrease in inter- and intra-kinetic variability in RT-qPCR is the incorporation of multiple stable reference genes [74]. 

Several algorithms have been developed in order to determine the most stable genes in specific experimental conditions, such as geNorm, NormFinder and BestKeeper. Following the identification of candidate reference genes, the geometric mean of their expression levels can be used to calculate the normalization factor [74].

### 4.3. Strengths and Limitations

This systematic review is the first one including 32 articles on circulating miRNAs as biomarkers in endometriosis. Our review provides an exhaustive overview of the 141 dysregulated miRNAs in endometriosis, with 13 miRNAs occurring in two studies and a small subset of ‘high-potential’ miRNAs described in three, (miR-20a-5p, miR-143-3p, miR-199a-5p and miR-3613-5p), four (miR-451a and let-7b) and six studies (miR-17-5p). Nevertheless, this study similarly has limitations which need to be mentioned. First of all, several included studies had rather small sample sizes in both cases and controls. Secondly, the majority of studies did not include an independent validation step, thereby potentially leading to false-positive results. Furthermore, a chance of selection bias and missed miRNAs exists as some studies did not establish a global profile of miRNAs but instead focused on preselected miRNAs previously mentioned in other studies. Finally, due to the heterogeneity between different studies (i.e., significant differences in study population and methodology for miRNA expression profiling), it was not feasible to perform a meta-analysis of the data.

## 5. Conclusions

To summarize, in our systematic review with 32 included articles, we detected the differential expression of 141 circulating miRNAs in endometriosis patients, with 20 miRNAs occurring in 2 or more studies. MiR-17-5p was found to be downregulated in six studies, with miR-451a and let-7b-5p in four studies, miR-20a-5p, miR-143-3p, miR-199a-5p and miR-3613-5p in three studies and thirteen other miRNAs in two studies. The lack of reproducibility between different studies is multifactorial and is the most important limitation of the current literature on circulating miRNAs in endometriosis. With laparoscopy as the only option to confirm or exclude endometriosis in symptomatic women with uncertain imaging, a need for a non-invasive test still exists. Based on the current literature, reviewed in this paper, individual miRNAs or panels of circulating miRNAs could have value as a biomarker in diagnosis and possibly even as future targets for novel treatments in endometriosis. To obtain further insights in this area, prospective studies with a large and well-described patient population, standardized protocols and an independent validation cohort are needed.

## Figures and Tables

**Table 1:** Included papers.

No	Author	Sample Size		Sample Type	ASRM Stage *	Control Group Characteristics	Methods	Normalization Control
1	Wang et al., 2013 [20]	Cases Controls	60 25	Serum	I–IV	NLP ILP: severe DM, PM, IF	1. Microarray 2. RT-qPCR	U6 snRNA
2	Hsu et al., 2014 [21]	Cases Controls	40 25	Serum	II–IV	NLP ILP: OBGP	1. Microarray 2. RT-qPCR	18s RNA
3	Cho et al., 2015 [22]	Cases Controls	24 24	Serum	III–IV	NLP ILP: PM, PP, SE, IF, OBGP	RT-qPCR	U6 snRNA
4	Cosar et al., 2016 [23]	Cases Controls	24 24	Serum	III–IV	NLP ILP: PM, PP, IF, SE	1. Microarray 2. RT-qPCR	U6 snRNA
5	Wang et al., 2016 [24]	Cases Controls	30 20	Serum	I–II	NLP ILP: PM, IF	1. Solexa sequencing 2. RT-qPCR	Cel-miR-39
6	Nothnick et al., 2017 [25]	Cases Controls	41 40	Serum	I–IV	n = 20: NLP ILP: PP, pain with bleeding n = 20: (SR)HW	RT-qPCR	U6 snRNA
7	Maged et al., 2018 [26]	Cases Controls	45 35	Serum	I–IV	NLP ILP: PP, IF, benign neoplasms	RT-qPCR	U6 snRNA
8	Hu et al., 2019 [27]	Cases Controls	20 26	Serum	III–IV	NLP or NLT ILP or ILT: PM, PP, IF, SE	RT-qPCR	U6 snRNA
9	Moustafa et al., 2020 [28]	Cases Controls	41 59	Serum	I–IV	NLP ILP: OBGP	RT-qPCR	U6 snRNA
10	Pang et al., 2020 [29]	Cases Controls	20 30	Serum	NS	Uterine fibroids, endometriosis free (NFS)	NS	NS
11	Misir et al., 2021 [30]	Cases Controls	71 65	Serum	I–IV	NLP or NLT	RT-qPCR	U6 snRNA
12	He et al., 2022 [31]	Cases Controls	23 20	Serum	I–IV	Tubal obstruction with NLP	RT-qPCR	U6 snRNA
13	Neuhausser et al., 2022 [32]	1. DC Cases Controls 2. VC Cases Controls	21 24 27 24	Serum	NS	Asymptomatic egg donors with normal pelvic US	1. NanoString nCounter 2. RT-qPCR	U6 snRNA
14	Kumari et al., 2022 [33]	Cases Controls	10 10	Serum	NS	NLP or NLT ILP: PP, SE, PM, IF, OBGP	RT-qPCR	miR-39
15	Lin et al., 2023 [34]	Cases Controls	80 80	Serum	I-IV	HW: normal gynecological examination and US	RT-qPCR	U6 snRNA
16	Yang et al., 2023 [35]	Cases Controls	17 13	Serum	NS	HT for uterine fibroids or CIN grade II-III	RT-qPCR	U6 snRNA
17	Suryawanshi et al., 2013 [36]	Cases Controls	33 20	Plasma	NS	SRHW	1. RT-qPCR 2. RT-qPCR	miR-132
18	Jia et al., 2013 [37]	Cases Controls	23 23	Plasma	III–IV	NLP ILP: PM, PP, IF, UL	1. Microarray 2. RT-qPCR	miR-16
19	Rekker et al., 2015 [38]	Cases Controls	61 65	Plasma	I–IV	n = 35: NLP ILP: SE, DM, IF, PP, polycystic ovaries n = 30: SRHW	RT-qPCR	miR-30e-5p, miR-99a-5p
20	Bashti et al., 2018 [39]	Cases Controls	55 23	Plasma	I–IV	NLP ILP: prolapsed uterus, ovarian cyst, urinary incontinence	RT-qPCR	miR-103-3p
21	Wang et al., 2018 [40]	Cases Controls	80 60	Plasma	I–IV	NLP ILP: SE, PM, PP, IF, UL	RT-qPCR	Beta-actin
22	Pateisky et al., 2018 [41]	Cases Controls	51 41	Plasma	I–IV	NLP ILP: SE, PP, IF, adnexal cysts, uterine fibroids	q-PCR-array based microarrays	miR-199a
23	Nisenblat et al., 2019 [42]	1.Cases Controls 2.a) Cases Controls 2.b) Cases Controls 3.Cases Controls 4.Cases Controls	8 8 8 8 10 10 51 27 80 39	Plasma	I–IV	1. Asymptomatic (SR)HW 2.a) Asymptomatic (SR)HW 2.b) NLP ILP: PP and/or IF 3. NLP ILP: DM, DP, chronic PP, IF 4. NLP ILP: DM, DP, chronic PP	1. Microarray 2. RT-qPCR	miR-30b
24	Vanhie et al., 2019 [43]	1. DC Cases Controls 2. VC Cases Controls	82 38 60 30	Plasma	I–IV	NLP ILP: surgical treatment of endometriosis diagnosed on imaging or diagnostic laparoscopy, PP with SE, IF	1. NGS 2. RT-qPCR 3. RT-qPCR	hsa-miR-423-3p, hsa-miR-28-3p
25	Hossein Razi et al., 2019 [44]	Cases Controls	25 25	Plasma	III–IV	NLP ILP: PP, ovarian cyst	1. NGS 2. RT-qPCR	SNORD47
26	Papari et al., 2020 [45]	Cases Controls	25 28	Plasma	III–IV	NLP ILP: PM, PM, IF, UL	RT-qPCR	hsa-miR-148b-3p, hsa-miR-30e-5p
27	Gu et al., 2020 [46]	Cases Controls	19 21	Plasma	Ovarian endometriosis, NS	HW (NFS)	RT-qPCR	NS
28	Zafari et al., 2021 [47]	Cases Controls	25 25	Plasma	III-IV	NLP ILP: PP, IF, DM	RT-qPCR	miR-16
29	Bahramy et al., 2021 [48]	Cases Controls	30 30	Plasma	III–IV	NLP ILP: PP, IF, DM	RT-qPCR	miR-16
30	Bendifallah et al., 2022 [49]	Cases Controls	153 47	Plasma	I–IV	Negative MRI and NLP ILP: OBGP, SE	NGS	NS
31	Tahermanesh et al., 2023 [50]	Cases Controls	30 30	Plasma	III-IV	HW with NLP	RT-qPCR	U6 snRNA
32	Walasik et al., 2023 [51]	Cases Controls	24 25	Plasma	III-IV	PM, OBGP, HW, NLP	RT-qPCR	miR-39

* Endometriosis stage in cases, according to the revised American Society for Reproductive Medicine (ASRM) classification [19]. Abbreviations: NLP = negative laparoscopy for endometriosis; NLT = negative laparotomy for endometriosis; ILP = indication for laparoscopy; ILT = indication for laparotomy; PP = pelvic pain; PM = pelvic mass; OBGP = other benign gynecological pathology; SE = suspected for endometriosis; IF = infertility; DM = dysmenorrhea; DP = dyspareunia; UL = uterine leiomyoma; EEC = endometrioid endometrial cancer; EOC = endometrioid ovarian carcinoma; US = ultrasonography; HW = healthy women; SRHW = self-reported healthy women; HT = hysterectomy; CIN = cervical intraepithelial neoplasia; OS = ovarian stimulation; NS = not specified; NFS = not further specified; NGS = next-generation sequencing; RT-qPCR = reverse transcription–quantitative polymerase chain reaction; U6 snRNA = U6 small nuclear RNA; DC = discovery cohort; VC = validation cohort.

**Table 2:** Significantly dysregulated microRNAs in two or more studies.

Serum			
No	Author	Dysregulated miRNAs	
1	Wang et al., 2013 [20]	↑	hsa-miR-122-5p, hsa-miR-199a-5p
		↓	hsa-miR-9-3p, hsa-miR-141-5p, hsa-miR-145-3p, hsa-miR-542-3p
2	Hsu et al., 2014 [21]	↓	hsa-miR-199a-5p
3	Cho et al., 2015 [22]	↓	hsa-let-7b-5p, hsa-mir-135a-5p
4	Cosar et al., 2016 [23]	↑	hsa-miR-18a-5p, hsa-miR-125b-5p, hsa-miR-143-3p, hsa-miR-150-5p, hsa-miR-342-3p, hsa-miR-451a, hsa-miR-500a-3p
		↓	hsa-miR-3613-5p, hsa-miR-6755-3p
5	Wang et al., 2016 [24]	↑	hsa-miR-185-5p, hsa-miR-424-3p
		↓	hsa-miR-15b-5p, hsa-miR-20a-5p, hsa-miR-30c-5p, hsa-miR-99b-5p, hsa-miR-127-3p
6	Nothnick et al., 2017 [25]	↑	hsa-miR-451a
7	Maged et al., 2018 [26]	↑	hsa-miR-122-5p, hsa-miR-199a-5p
8	Hu et al., 2019 [27]	↓	hsa-miR-370-3p
9	Moustafa et al., 2020 [28]	↑	hsa-miR-125b-5p, hsa-miR-150-5p, hsa-miR-342-3p, hsa-miR-451a
		↓	hsa-let-7b-5p, hsa-miR-3613-5p
10	Pang et al., 2020 [29]	↓	hsa-miR-17-5p
11	Misir et al., 2021 [30]	↑	hsa-miR-200c
		↓	hsa-miR-34a-5p
12	He et al., 2022 [31]	↓	hsa-mir-148a
13	Neuhausser et al., 2022 [32]	DC ↑	hsa-miR-10a-5p, hsa-miR-182-3p, hsa-miR-210-5p, hsa-miR-219a-5p, hsa-miR-363-3p, hsa-miR-478a-3p, hsa-miR-495-3p, hsa-miR-513b-5p, hsa-miR-518c-3p, hsa-miR-626, hsa-miR-802, hsa-miR-942-5p, hsa-miR-1228-3p, hsa-miR-1249-3p, hsa-miR-1266-5p, hsa-miR-1306-5p, hsa-miR-4443 and hsa-miR-4516
		DC ↓	hsa-miR-34c-3p
		VC ↓	hsa-miR-34c-3p
14	Kumari et al., 2022 [33]	↑	hsa-miR-99b-5p, hsa-miR-125a-5p, hsa-miR-143-3p, hsa-miR-145-5p
		↓	hsa-miR-16
15	Lin et al., 2023 [34]	↓	hsa-miR-17-5p, hsa-miR-424-5p
16	Yang et al., 2023 [35]	↓	hsa-miR-17-5p
Plasma			
17	Suryawanshi et al., 2013 [36]	↑	hsa-miR-15b-5p, hsa-miR-16-5p, hsa-miR-191-5p, hsa-miR-195-5p, hsa-miR-362-5p, hsa-miR-1973, hsa-miR-1974, hsa-miR-1978, hsa-miR-1979, hsa-miR-4284
18	Jia et al., 2013 [37]	↓	hsa-miR-17-5p, hsa-miR-20a-5p, hsa-miR-22-5p
19	Rekker et al., 2015 [38]	↓	hsa-mir-141-3p, hsa-mir-200a-3p
20	Bashti et al., 2018 [39]	↑	hsa-miR-145-5p
		↓	hsa-miR-31-5p
21	Wang et al., 2018 [40]	↓	hsa-miR-17-5p
22	Pateisky et al., 2018 [41]	↑	hsa-miR-33a-5p
		↓	hsa-miR-154-5p, hsa-miR-196b-5p, hsa-miR-378a-3p
23	Nisenblat et al., 2019 [42]	DC ↑	hsa-miR-145-3p
		DC ↓	hsa-miR-9-3p, hsa-miR-135b-5p, hsa-miR-139-3p, hsa-miR-141-3p, hsa-miR-155-5p, hsa-miR-574-3p, hsa-miR-923
		VC ↑	No differentially expressed miRNAs
		VC ↓	hsa-miR-139-3p, hsa-miR-155-5p, hsa-miR-574-3p
24	Vanhie et al., 2019 [43]		No differentially expressed miRNAs
25	Hossein Razi et al., 2019 [44]	↓	hsa-miR-185-5p
26	Papari et al., 2020 [45]	↓	hsa-let-7b-5p, hsa-miR-17-5p, hsa-miR-20a-5p, hsa-miR-21-5p, hsa-miR-103a-3p, hsa-miR-143-3p, hsa-miR-199a-3p, hsa-miR-340-5p
27	Gu et al., 2020 [46]	↓	hsa-let-7a-5p, hsa-let-7b-5p, hsa-let-7d-5p, hsa-let-7f-5p, hsa-let-7g-5p, hsa-let-7i-5p, hsa-miR-199a-3p, hsa-miR-320a, hsa-miR-320b, hsa-miR-320c, hsa-miR-320d, hsa-miR-328-3p, hsa-miR-131-3p, hsa-miR-320e
28	Zafari et al., 2021 [47]	↑	hsa-miR-199b-3p
		↓	hsa-let-7d-3p, hsa-miR-224-5p
29	Bahramy et al., 2021 [48]	↓	hsa-miR-92a-3p, hsa-miR-340-5p, hsa-miR-381-3p
30	Bendifallah et al., 2022 [49]	↑	hsa-miR-29b-1-5p, hsa-miR-3122, hsa-miR-4536-3p, hsa-miR-4715-5p, hsa-miR-6502-5p
		↓	hsa-miR-203a-5p, hsa-miR-208a-5p, hsa-miR-208a-3p, hsa-miR-216b-3p, hsa-miR-504-3p, hsa-miR-514b-5p, hsa-miR-573, hsa-miR-889-5p, hsa-miR-1180-5p, hsa-miR-1253, hsa-miR-1910-5p, hsa-miR-1973, hsa-miR-3064-3p, hsa-miR-3137, hsa-miR-3168, hsa-miR-3185, hsa-miR-3622a-3p, hsa-miR-3923, hsa-miR-4674, hsa-miR-4703-5p, hsa-miR-4725-5p, hsa-miR-4740-5p, hsa-miR-4749-5p, hsa-miR-4750-3p, hsa-miR-4764-5p, hsa-miR-5004-3p, hsa-miR-6075, hsa-miR-6811-3p, hsa-miR-6824-3p, hsa-miR-6875-3p, hsa-miR-6788-3p, hsa-miR-6799-3p, hsa-miR-7108-3p, hsa-miR-7109-5p, hsa-miR-7150, hsa-miR-7152-5p
31	Tahermanesh et al., 2023 [50]	↑	hsa-miR-490-3p, hsa-miR-1271-5p
32	Walasik et al., 2023 [51]	↓	hsa-miR-451a, hsa-miR-3613-5p

Legend: miRNAs written in blue or red font are respectively consistently up- or downregulated in two or more studies. miRNAs written in orange font represent miRNAs described in two or more studies with opposite results. Abbreviations: UV = univariate; MV = multivariate; DC = discovery cohort; VC = validation cohort.
